# Risk Factors for Non-Space-Occupying Postoperative Hemorrhages Following Brain Tumor Resection Without the Influence of Anticoagulant or Antiplatelet Therapy: A Ten-Year Single-Center Retrospective Analysis

**DOI:** 10.3390/neurolint18020030

**Published:** 2026-02-09

**Authors:** Anatoli Pinchuk, Nikolay Tonchev, Anna Schaufler, Claudia A. Dumitru, Belal Neyazi, Klaus-Peter Stein, I. Erol Sandalcioglu, Ali Rashidi

**Affiliations:** Department of Neurosurgery, Otto-von-Guericke-University, 39120 Magdeburg, Germanynikolay.tonchev@med.ovgu.de (N.T.); belal.neyazi@med.ovgu.de (B.N.); erol.sandalcioglu@med.ovgu.de (I.E.S.)

**Keywords:** brain tumors, non-space-occupying cerebral hemorrhage, clinical characteristics

## Abstract

**Background/Objectives**: Postoperative intracerebral hematomas (POHs) are a common complication following brain tumor surgery and are typically associated with unfavorable outcomes. While extensive hemorrhages have been studied extensively, smaller, Non-Space-Occupying hemorrhages are frequently detected, yet their clinical relevance and associated risk factors remain insufficiently understood. This study aimed to identify predictive factors for the occurrence of Non-Space-Occupying postoperative cerebral hemorrhages in patients undergoing brain tumor resection. **Methods**: A total of 1481 patients without a history of anticoagulant or antiplatelet therapy underwent brain tumor surgery at our neurosurgical institute over a ten-year period. Non-Space-Occupying postoperative hemorrhages were diagnosed in 84 patients using cranial computed tomography (cCT) or magnetic resonance imaging (cMRI) performed after the tumor resection. Demographic data, pre-existing comorbidities, and tumor characteristics were collected and analyzed. **Results**: Non-Space-Occupying POHs occurred in 5.6% of patients. The most frequent tumor type associated with POHs was glioblastoma multiforme (N = 33; 39.3%), followed by metastatic lesions (N = 9; 10.7%) and benign primary intracranial neoplasms (N = 31; 38%). None of the affected patients exhibited new neurological deficits or signs of increased intracranial pressure. A multivariate analysis identified the tumor size as an independent risk factor for Non-Space-Occupying POHs (*p* = 0.002), with patient age emerging as the strongest predictor (*p* = 0.001). **Conclusions**: Non-Space-Occupying POHs after a brain tumor resection are significantly associated with the tumor size, an advanced patient age, and the presence of pre-existing liver disease. The recognition of these risk factors may facilitate targeted perioperative monitoring and guide postoperative management strategies.

## 1. Introduction

Intracranial tumors such as glioblastoma multiforme, meningiomas, and cerebral metastases represent a major clinical challenge, and surgical resection remains a cornerstone in their treatment. The potential benefits of brain tumor resection include the alleviation of neurological symptoms, a reduction in tumor mass to facilitate subsequent adjuvant treatments, and the extension of the overall survival rate [1,2,3,4,5]. Despite these advantages, cranial neurosurgical procedures carry inherent risks. Among these, a postoperative intracranial hemorrhage (POH), although relatively uncommon, is one of the most feared complications due to its potential to cause severe neurological deterioration or even death. Major postoperative hematomas requiring surgical evacuation are rare, with reported incidence rates between 1% and 3% [1,2,3,4,5].

In contrast to major hematomas, smaller amounts of postoperative blood—often identified adjacent to the surgical approach or within the resection cavity—are encountered more frequently, yet their clinical relevance is not well defined. These small hemorrhages can typically be visualized on early postoperative cranial MRI or CT as hyperintense foci, reflecting the presence of methemoglobin within the extravasated blood. The lack of a necessity for a specific treatment, as well as the absence of standardized follow-up protocols, makes the systematic investigation of these hemorrhages difficult. Nevertheless, in most cases, the appearance of such bleeding on postoperative imaging leads to repeated radiological examinations and closer clinical monitoring.

The pathophysiology of POHs following brain tumor resection is multifactorial. Common mechanisms include persistent oozing within the surgical cavity due to incomplete hemostasis, subdural hematoma formation secondary to traction on cortical vessels, and parenchymal contusion resulting from the excessive retraction necessitated by limited surgical exposure [6,7,8]. Previous investigations into gliomas have shown that early postoperative cMRI detects blood in or near the resection cavity in approximately 24% of cases and extra-axial hemorrhages in about 6% [9], while a smaller cohort reported blood in all examined cases [10]. Studies of larger POHs have identified an advanced patient age [3,5] and the tumor size [3] as potential risk factors. However, these findings cannot be directly extrapolated to small, Non-Space-Occupying hemorrhages. Furthermore, the possible clinical implications of such smaller hemorrhages remain unclear, as they typically do not produce overt neurological deficits. Nevertheless, extravasated blood has been shown to exert neurotoxic effects and may be associated with adverse cognitive outcomes [11].

Given these uncertainties, a focused investigation of small, Non-Space-Occupying POHs becomes relevant. The present study was designed to systematically evaluate all cases of Non-Space-Occupying POHs detected in or near the resection cavity on early postoperative cranial MRI or CT in patients without the confounding effects of anticoagulant or antiplatelet therapy. The primary objective was to identify predictive risk factors for the first occurrence of a POH in patients undergoing surgical treatment for both malignant and benign brain tumors. A secondary objective was to assess the influence of the preoperative tumor volume, as a morphological parameter, on the occurrence of hemorrhagic transformation following tumor resection.

## 2. Materials and Methods

### 2.1. Study Design and Setting

This retrospective observational study was conducted at the Department of Neurosurgery at our institution. All consecutive patients who underwent intracranial tumor resection between January 2008 and December 2018 were eligible for inclusion. Data collection was performed between February and November 2019.

### 2.2. Participants

Patients were included if they underwent surgical resection of an intra- or extra-axial intracranial tumor during the study period. Individuals receiving anticoagulant or antiplatelet therapy at the time of surgery were excluded to avoid confounding effects on the risk of postoperative hemorrhage (POH). A total of 1481 patients met the inclusion and exclusion criteria. Patients with poor documentation of the course of disease were excluded from this study.

### 2.3. Outcome Definition and Follow-Up

The primary outcome was the occurrence of a Non-Space-Occupying postoperative hemorrhage, identified via routine postoperative imaging. In accordance with institutional protocols, all patients underwent cranial CT or MRI within 48 h after surgery. Non-Space-Occupying hemorrhage was defined as any intracerebral or extra-axial blood collection located in or adjacent to the resection cavity that did not produce mass effect, neurological deficits, or midline shift and did not require surgical intervention. Follow-up duration varied among patients, ranging from two months to several years.

### 2.4. Data Sources and Variables

Clinical data extracted from the institutional database included demographic parameters, such as age, sex, medical history, and comorbidities, with a particular focus on cardiovascular disease, diabetes mellitus, coagulopathies, and liver disease. This information was assessed based on medical documentation and past medical history reports. Tumor characteristics were determined from preoperative imaging and histopathological reports, including tumor entity, location, and tumor volume calculated via contrast-enhanced T1-weighted MRI sequences. These calculations were carried out based on preoperative MRI, using SmartBrush volumetric software as a part of Brainlab Elements^®^ Version 3.0 (Brianlab AG, Munich, Germany).

### 2.5. Surgical and Perioperative Factors

Surgical technique and duration of surgery were not included in the analysis due to the heterogeneity of pathological entities and the inclusion of both intra- and extra-axial tumors, which limited comparability across procedures.

### 2.6. Bias and Study Size

The use of consecutive cases and a standardized postoperative imaging protocol minimized selection and detection bias, particularly for clinically silent hemorrhages. The study size was determined by the number of eligible patients treated during the predefined 11-year period.

### 2.7. Statistical Analysis

Descriptive statistics were used to summarize baseline characteristics. Categorical variables were analyzed using the chi-square test or Fisher’s exact test, and continuous variables were compared using Student’s *t*-test or Mann–Whitney U test, depending on data distribution. Variables with *p* < 0.05 in univariate analysis were entered into a multivariate logistic regression model to identify independent predictors of Non-Space-Occupying postoperative hemorrhage. Statistical significance was set at *p* < 0.05. Analyses were performed using R version 4.5.1 incorporating the packages gtsummary version 2.3.0 for statistical testing and ggplot2 version 3.5.2 as well as gghalves version 0.1.4 for visualization.

## 3. Results

During the study period, a total of 1481 patients underwent an intracranial tumor resection. POHs occurred in 106 patients, corresponding to an incidence of 7.16% (Table 1).

Of these, 84 patients (5.6%) developed Non-Space-Occupying POHs (Table 2).

**Figure 1:**
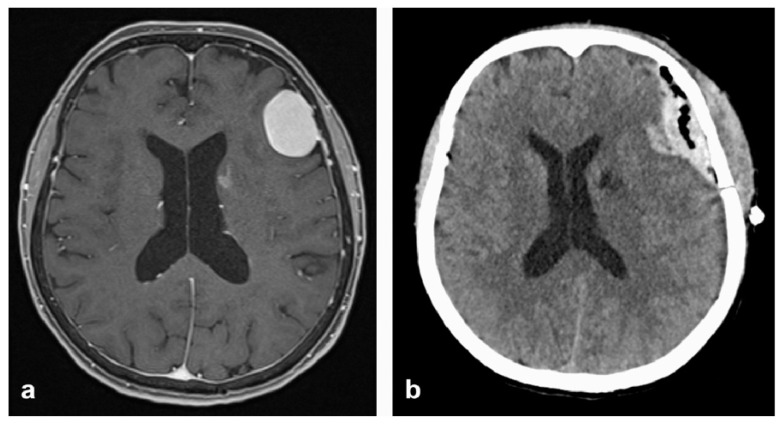
MRI (**a**) and CT (**b**) axial scans of head. Example of postoperative hemorrhage in the cavity after resection of a convexity meningioma.

Figure 1 shows an example of the individual bleeding location in postoperative cCT examinations.

### 3.1. Predictors of Hemorrhagic Complications

Potential predictors of hemorrhagic complications were evaluated in the entire study cohort. Initially, univariate analyses were performed to examine the influence of demographic variables, pre-existing medical conditions, and tumor characteristics (Table 3).

### 3.2. Demographic Characteristics

**Figure 2:**
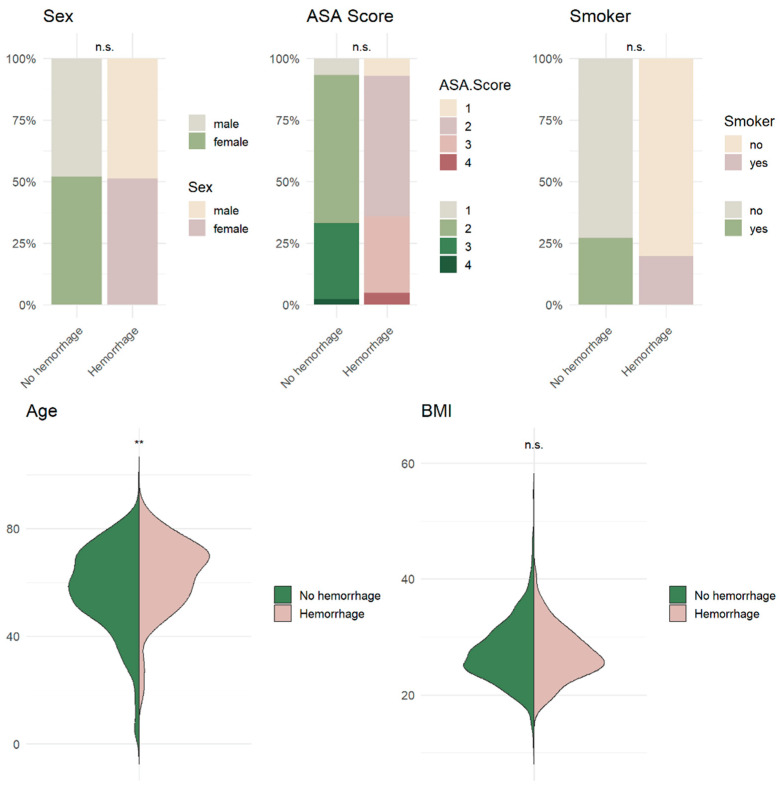
Graphic visualization of the relevant demographic parameters with potential significance in the context of postoperative hemorrhage following cranial surgery.

**Figure 3:**
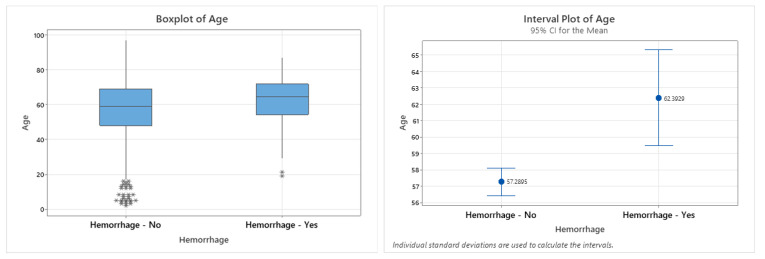
Graphical presentation of the patient distribution according to age, employing both box plots and interval plots. The diagrams illustrate the minimum, median, and maximum values among both groups: patients with and without hemorrhage.

Among the 84 patients with Non-Space-Occupying POHs, 41 were male (48.8%) and 43 were female (51.2%), with a mean age of 62.4 years (±13.5). No statistically significant associations were observed between the hemorrhage occurrence and sex (*p* = 0.885), smoking status (*p* = 0.150), body mass index (BMI) (*p* = 0.424), or American Society of Anesthesiologists (ASA) physical status classification (*p* = 0.538). In contrast, the patient age at the time of surgery demonstrated a statistically significant association with the occurrence of hemorrhages (*p* = 0.001) (Figure 2 and Figure 3, Table 3).

### 3.3. Comorbidities

**Figure 4:**
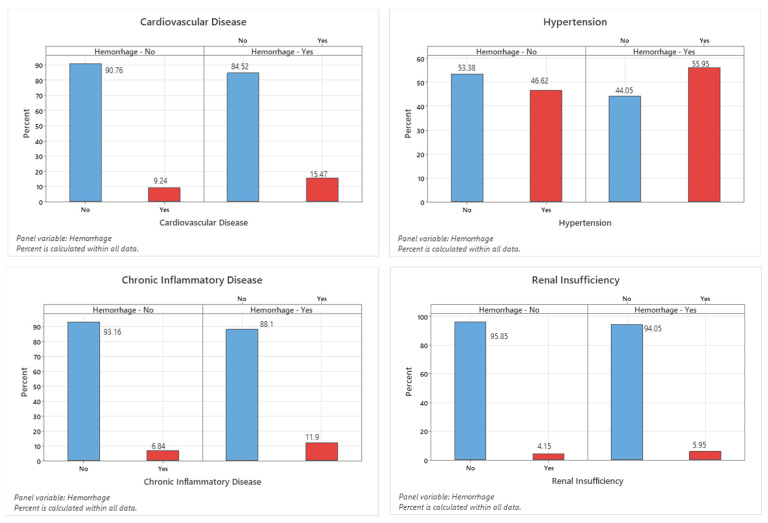
Graphic visualization of the relevant pre-existing conditions with potential significance in the context of postoperative hemorrhage following cranial surgery.

In the univariate analysis, arterial hypertension (*p* = 0.096), diabetes mellitus (*p* = 0.500), cardiac disease (*p* = 0.059), renal insufficiency (*p* = 0.425), and chronic inflammatory conditions (*p* = 0.080) were not significantly associated with the risk of postoperative hemorrhages. In contrast, the presence of a pre-existing liver disease demonstrated a statistically significant correlation with hemorrhage occurrence (*p* = 0.028) (Figure 4 and Table 4).

### 3.4. Tumor Characteristics

**Figure 5:**
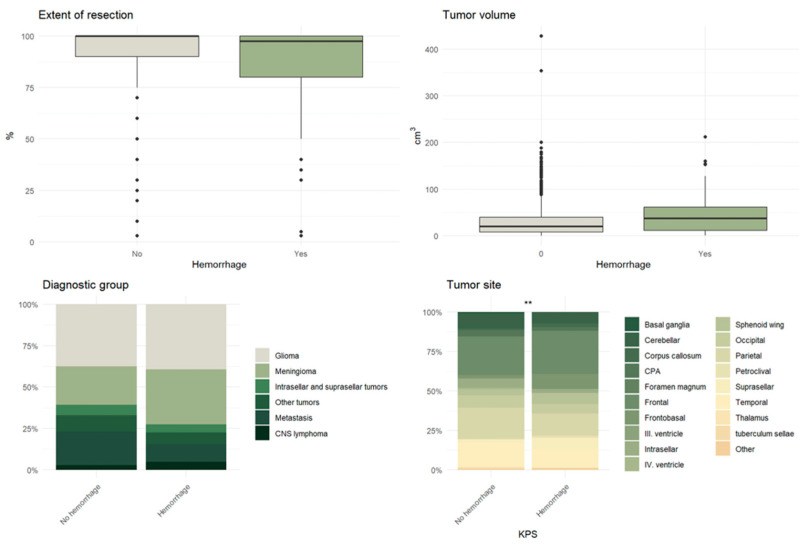
Graphic visualization of the relevant tumor characteristics (tumor volume, histopathological type, tumor site and extent of tumor resection) with potential significance in the context of postoperative hemorrhage following cranial surgery.

**Figure 6:**
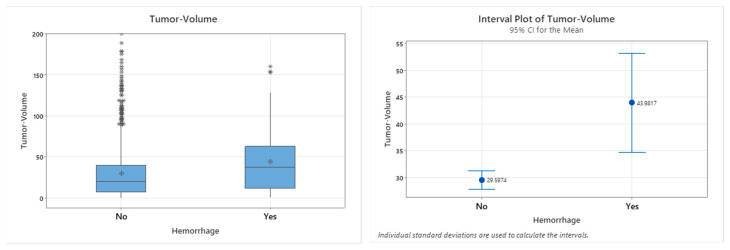
Graphical presentation of the patient distribution according to tumor volume, employing both box plots and interval plots. The diagrams illustrate the minimum, median, and maximum values among both groups: patient with and without hemorrhage.

Among the 84 patients with Non-Space-Occupying POHs, 33 had glioblastoma, 28 had meningioma, 9 had metastatic lesions, 4 had intra- or suprasellar tumors, and 4 had primary central nervous system lymphoma (Table 5). Tumor characteristics, including the tumor type (*p* = 0.097) and side (*p* = 0.640), did not show statistically significant associations with postoperative hemorrhages. Apart from these parameters, no further significant correlations were identified. In contrast, the tumor volume demonstrated a statistically significant association with the occurrence of hemorrhages (*p* = 0.002) (Table 5, Figure 5 and Figure 6).

## 4. Discussion

In this retrospective analysis of 1481 patients, 84 developed Non-Space-Occupying postoperative cerebral hemorrhages, all occurring in the absence of anticoagulants or antiplatelet therapy. We identified distinct clinical and tumor-related factors associated with these hemorrhagic events. Our findings demonstrated that traditional vascular risk factors, such as arterial hypertension, diabetes mellitus, smoking history, and coronary heart disease, were not significantly associated with the occurrence of postoperative hemorrhages. This observation aligns with previous reports suggesting that such vascular risk factors may play a less prominent role in the pathogenesis of postoperative cerebral bleeding in patients with malignant diseases [12,13,14,15]. Among the 1481 patients who underwent intracranial tumor resections, postoperative intracranial hemorrhages occurred in 106 patients (7.16%). Of these, 84 patients (5.6%) developed Non-Space-Occupying postoperative hemorrhages. These lesions were detected radiologically within 48 h post-surgery and did not cause mass effects or require surgical evacuation.

In the hemorrhage group, 41 patients (48.8%) were male and 43 (51.2%) were female, with a mean age of 62.4 ± 13.5 years. Their sex, smoking status, BMI, and ASA physical status classification were not significantly associated with the hemorrhage risk. However, an advanced age at the time of surgery was a strong predictor (*p* = 0.001). The age association aligns with previous reports linking older ages to an increased risk of postoperative hematomas [3,6], possibly due to age-related vascular fragility, impaired autoregulation, and a reduced hemostatic reserve. Arterial hypertension, diabetes mellitus, cardiac disease, renal insufficiency, and chronic inflammation showed no significant association with postoperative bleeding. In contrast, pre-existing liver diseases correlated significantly with hemorrhage occurrence (*p* = 0.028). This finding likely reflects the subtle coagulopathies and platelet dysfunction observed in liver disease, even without overt clinical signs, and supports the need for heightened perioperative vigilance in this subgroup [16,17,18].

The histopathological analysis showed that hemorrhages occurred most frequently in glioblastoma (n = 33), followed by meningioma (n = 28), metastases (n = 9), intra-/suprasellar tumors (n = 4), and primary CNS lymphoma (n = 4). Neither the tumor type (*p* = 0.097) or laterality (*p* = 0.640) were significantly associated with hemorrhages, and the WHO grade was not predictive of the bleeding risk. These results are consistent with prior studies indicating that the tumor location, histology, and grade are not independent predictors of postoperative hematomas [3,5,19]. While malignant gliomas and metastases are biologically predisposed to bleeding due to angiogenesis and neovascularization [20,21,22], our data suggests that these features do not independently predict Non-Space-Occupying postoperative hemorrhages. Meningioma-associated hemorrhages, although less common in general, may relate to tumor vascular malformations [23]. In our study the tumor volume was a significant independent predictor of hemorrhages (*p* = 0.002). Larger tumors create more extensive resection cavities, increasing the surface area available for postoperative oozing and the potential for intradural collections. In the case of extradural hemorrhages, a dural collapse into the cavity after closure may open space for hematoma formation. Larger tumors also typically necessitate larger craniotomies, which could further contribute to the risk [3,24,25]. This finding mirrors previous work establishing tumor size as a key risk factor for clinically significant postoperative hematomas [3] and suggests that similar mechanisms may underlie the formation of smaller hemorrhages.

No significant association was found between perioperative hypertension and Non-Space-Occupying postoperative hemorrhages in our series. This contrasts with some studies linking hypertension to hematomas requiring reoperation [3,26,27], suggesting that mechanisms leading to large hematomas may differ from those for smaller, imaging-only lesions. Although the hemorrhages observed in this study were radiologically detectable but clinically silent, experimental and clinical data suggest that even small amounts of extravasated blood can exert neurotoxic effects and may contribute to subtle cognitive or functional deficits [11]. The potential impact may be most relevant in patients with large tumors, where both the resection cavity size and blood volume are greater. The literature on the relationship between the tumor size and postoperative cognitive change is mixed, with some studies reporting an association [28] and others finding none [29,30].

The strengths of this study include a targeted exclusion of patients receiving anticoagulant or antiplatelet therapy, a systematic imaging review, and the inclusion of both benign and malignant tumors, which increases the external validity. The diagnosis of Non-Space-Occupying POHs leads often to multiple follow-up radiological imaging tests, which increases treatment costs. In the absence of neurological symptoms, these precautionary measures should be limited to high-risk patients. Therefore, investigating such risk factors becomes important for ensuring proper patient selection and postoperative care.

The limitations of the current study include its retrospective design, potential selection bias, and the absence of long-term neurocognitive follow-ups. Another important limitation is the lack of detailed, quantifiable data on the surgical technique and surgical approach. Because this study spans more than a decade, operations were performed by multiple senior surgeons with varying levels of experience, individual preferences, and evolving surgical strategies. This heterogeneity may have introduced variability in the intraoperative hemostasis, specific microsurgical technique, and tissue manipulation, all of which could influence the occurrence of postoperative hemorrhages.

Furthermore, this study did not incorporate systematic postoperative neurological or cognitive assessments, limiting the conclusions regarding the functional significance of these radiological findings. Finally, although patients receiving anticoagulant or antiplatelet therapy were excluded to reduce confounding, unmeasured factors such as intraoperative blood pressure fluctuations, subtle coagulation abnormalities, or surgeon-specific thresholds for preoperative hemostasis parameters may still have influenced the results.

## 5. Conclusions

Non-Space-Occupying postoperative intracranial hemorrhages emerged as a relatively frequent radiological finding following intracranial tumor resection, occurring in 5.6% of all patients in our cohort. Within the broader group of postoperative hemorrhages (7.16%), these smaller, clinically silent lesions represented the majority of cases. Despite the multifactorial nature of postoperative bleeding, our analysis identified several distinct predictors of Non-Space-Occupying POHs. A larger preoperative tumor volume, advanced patient age, and pre-existing liver disease were significantly associated with hemorrhage occurrence, underscoring the relevance of both morphological and systemic factors in the postoperative bleeding risk.

In contrast, traditional vascular risk factors—including arterial hypertension, diabetes mellitus, cardiovascular disease, renal insufficiency, and chronic inflammatory conditions—did not demonstrate significant associations. Similarly, demographic variables such as sex, the BMI, and smoking status were not predictive. Tumor-related characteristics, including the tumor type, WHO grade, and tumor location, also showed no significant correlation with the hemorrhage risk. These findings suggest that the development of Non-Space-Occupying POHs is driven less by classical vascular comorbidities or tumor histology and more by structural and age-related vulnerability.

The recognition of these risk factors may help identify patients at increased risk, guide perioperative monitoring strategies, and inform surgical planning—particularly in cases involving large tumors or patients with underlying hepatic dysfunction. Although the hemorrhages observed were radiologically detectable but clinically silent, their potential neurobiological impact remains uncertain. Future prospective studies incorporating standardized postoperative neuroimaging and structured neurocognitive follow-up are warranted to clarify the functional and quality-of-life implications of these small postoperative hemorrhages.

## Figures and Tables

**Table 1:** Distribution of patients according to history of bleeding.

		N	%
**Hemorrhage**	Yes	106	7.16
	No	1375	92.84

**Table 2:** Distribution of patients with POH with and without surgery.

		N	%
**Hemorrhage**	No	1375	92.84
	Bleeding without surgery	84	5.67
	Bleeding with surgery	22	1.49

**Table 3:** Overview of patients exhibiting postoperative bleeding that does not necessitate reoperation.

			No Hemorrhage N (%), Mean ± SD	Hemorrhage N (%), Mean ± SD	*p*-Value
**Demographic parameters**	Sex	Female Male	715 (52.0) 660 (48.0)	143 (51.19) 41 (48.81)	0.885
	Age		57.3 ± 15.9	62.4 ± 13.5	**0.001**
	BMI		27.37 ± 5.5	26.97 ± 4.38	0.424
	ASA Score	I	93 (6.8)	6 (7.14)	0.538
		II	821 (60.01)	48 (57.14)	
		III	423 (30.92)	26 (30.95)	
		IV	31 (2.27)	4 (4.76)	
	Smoker	Yes No	364 (27.02) 983 (72.98)	16 (19.75) 65 (80.25)	0.150

**Table 4:** Overview table of comorbidities that potentially influence postoperative bleeding following cranial surgery. Liver disorders showed statistical correlation with postoperative bleeding complications.

			No Hemorrhage N, (%)	Hemorrhage N, (%)	*p*-Value
**Comorbidity**	Hypertension	Yes No	641 (46.62) 734 (53.38)	47 (55.95) 37 (44.05)	0.096
	Diabetes type 1/2	Yes No	208 (15.13) 1167 (84.87)	15 (17.86) 69 (82.14)	0.500
	Cardiovascular disease	Yes No	127 (9.24) 1248 (90.76)	13 (15.48) 71 (84.52)	0.059
	Chronic inflammation	Yes No	94 (6.84) 1281 (93.16)	10 (11.9) 74 (88.1)	0.080
	Renal failure	Yes No	57 (4.15) 1318 (95.85)	5 (5.95) 79 (94.05)	0.425
	Liver disorder	Yes No	30 (2.18) 1345 (97.82)	5 (5.95) 79 (94.05)	**0.028**

**Table 5:** Overview table of various tumor characteristics that potentially influence postoperative bleeding following cranial surgery. A strong statistical correlation has been demonstrated between tumor volume and postoperative hemorrhage among the study cohort.

			No Hemorrhage N (%), Mean	Hemorrhage N (%), Mean	*p*-Value
**Tumor characteristics**	Tumor type	Glioma Meningioma Metastases Intra- and suprasellar tumors CNS lymphoma Others	516 (37.53) 319 (23.2) 279 (20.29) 86 (6.25) 37 (2.69) 138 (10.04)	33 (39.29) 28 (33.33) 9 (10.71) 4 (4.76) 4 (4.76) 6 (7.14)	0.097
	Brain hemisphere	Left Midline Right	604 (43.93) 198 (14.4) 573 (41.67)	37 (44.05) 15 (17.86) 32 (38.1)	0.640
	Tumor volume (cm^3^)		22.84	34.45	**0.002**

## Data Availability

The original contributions presented in this study are included in the article. Further inquiries can be directed to the corresponding author.

## Abbreviations

The following abbreviations are used in this manuscript:

ASA	American Society of Anesthesiologists
BMI	body mass index
cCT	cranial computed tomography
cMRI	cranial magnetic resonance imaging
POH	postoperative intracerebral hematoma
